# Supplementary material for: Precision Nutrition Opportunities to Help Mitigate Nutrition and Health Challenges in Low- and Middle-Income Countries: An Expert Opinion Survey
Source: Nutrients. 2023 Jul 21;15(14):3247. doi: 10.3390/nu15143247 (PMC10385361; doi:10.3390/nu15143247)
Supplement: Supplementary file 1 [file nutrients-15-03247-s001.zip › nutrients-2455185-supplementary/Supplemental and annex/Figure S1.pdf]

SWISS FOOD & NUTRITION VALLEY

Agenda

1:00pm	**Introductory Session**		
	**Welcome**<br>*SFNV working group:*<br>Christina Senn-Jakobsen	**SFNV impact pot 4**<br>*SFNV working group:*<br>Christian Nils Schwab	**Precision Nutrition for LMIC working group learnings**<br>*SFNV working group:*<br>Dr Jacquelyn Bedsaul
1:20pm	**3-minute presentations:**<br>**Nutrition challenges and precision nutrition opportunities**<br>*Participants*		**Fireside Q&A**<br>*SFNV working group:*<br>Dr Klaus Kraemer
1:40pm	**Brainstorming Session**		
	**Precision Nutrition - collection and analysis of dietary intake and nutritional status data**		**Precision Nutrition - nutritional products, supplements, and treatments**
2:40pm	**Closing Session**		
	**Brainstorming session feedback**<br>*Moderators*	**Q&A**<br>*SFNV working group:*<br>Dr Diane Clayton	
2:55pm	**Next steps toward proof-of-concept**<br>*SFNV working group*<br>Dr Klaus Kraemer	**Final remarks**<br>*SFNV working group:*<br>Christina Senn-Jakobsen	Precision Nutrition for LMIC Virtual Workshop<br>30 November 2022 | 1:00 – 3:00 pm CET

Figure S1. Precision Nutrition for LMIC Virtual Workshop agenda. The workshop event took place on November 30, 2022 from 1-3:00 pm CET. The introductory session consisted of a welcome, introduction to the SFNV impact pot 4 group mission, as well as the precision nutrition (PN) for LMIC project learnings. Three-minute presentations were conducted by expert participants and a fireside question and answer (Q&A) was led by the SFNV working group. A brainstorming session was facilitated in breakout rooms on two topics 1) precision nutrition – collection and analysis of dietary intake and nutritional status data and 2) precision nutrition – nutritional products, supplements and treatments. The closing session consisted of the brainstorming session feedback and Q&A, next steps toward proof-of-concept, and final remarks by the SFNV working group.
