# Supplementary material for: Precision Nutrition Opportunities to Help Mitigate Nutrition and Health Challenges in Low- and Middle-Income Countries: An Expert Opinion Survey
Source: Nutrients. 2023 Jul 21;15(14):3247. doi: 10.3390/nu15143247 (PMC10385361; doi:10.3390/nu15143247)
Supplement: Supplementary file 1 [file nutrients-15-03247-s001.zip › nutrients-2455185-supplementary/Supplemental and annex/Tables S1, S2.pdf]

Table S1. Precision Nutrition for Low- and Middle-Income Countries expert opinion survey questionnaire.

**Questionnaire**
1. Please share with us your overall viewpoint on Precision Nutrition and any initial comments you may have. What is your current understanding of Precision Nutrition and where does your understanding stem from?
**Part I: The Problem**
2. Can you briefly summarize the most pressing nutrition challenges impacting populations in your region and the root cause of these challenges?
3. What are the segments or sub-segments of the population where you have identified these nutrition-related problems?
4. If you were to choose a target population to support from a nutritional point of view, which one would that be? How do you come to that conclusion?
5. What are the key barriers/challenges to address nutrition-related problems in your region (or the region you work in)?
**Part II - Current Solutions**
6. Where is information on the nutritional status of the population in the region available? Who/which stakeholder(s) is driving the need for this information and how is it primarily funded?
7. Which methods/approaches do you use to address the earlier mentioned challenges?
8. What efforts (research, implementation, strategies, resources etc...) are ongoing or in the pipeline to adapt and improve these methods?
9. What results are you seeing and which key challenges remain to be addressed?
10. How satisfied are you with the current methods, results, and overall progress in addressing the earlier mentioned nutrition-related challenges?
**Part III Precision Nutrition**
11. How helpful do you find this Precision Nutrition definition in your work?
12. Do you see a similar understanding of Precision Nutrition across the region (or the region you work in)? Please elaborate.
13. Within your research team and/or among other academic colleagues, what key areas of research have been identified for the coming 5 years? Has Precision Nutrition been mentioned or could Precision Nutrition support any of these research needs/priorities?
14. Are you aware of any research groups that are carrying out Precision Nutrition research in your country (or countries you work in)? What is their research on? Can you please name the institutions/research groups?

15. Are you aware of any ongoing Precision Nutrition solution(s) in your country (or countries you work in)? If so, please elaborate.

16. Do you see Precision Nutrition as a part of the solution to address the nutritional problems you mentioned earlier? Please elaborate.

17. Can you suggest 3 potential Precision Nutrition related approaches that could address the key nutritional challenges you have identified.

18. Do you see enablers and barriers for the implementation of Precision Nutrition solutions in your country (or countries you work with)? If so, what are they?

Part IV: Collaboration

19. Do you have past experience working in academic partnerships, notably with Swiss entities?

20. Do you regard Switzerland as a potential and attractive partner for a joint research program?

21. Would you be open to enter a joint collaboration with us around the topic of Precision Nutrition?

22. If yes, how would you envision this collaboration? What would you bring to the partnership, and what would you expect from the Swiss entities?

23. Do you work with any other international institutions (NGOs, funder, non-profit, start-up, private sector, research institutions) on similar topics? If so, please provide names/institutions.

24. Whom else should we definitely talk to in the context of Precision Nutrition and our discussion?

Table S2. Precision Nutrition for Low- and Middle-Income Countries Virtual Workshop brainstorming session discussion questions.

**Discussion Questions**
**Topic I: Precision Nutrition - collection and analysis of dietary intake and nutritional status data**
1. What efforts are currently being practiced around the collection and analysis of dietary intake and nutrition status data in LMIC? What is missing from current efforts to solve this challenge?
2. Which precision nutrition tool or approach could help mitigate these challenges?
3. What are the key steps that are necessary to turn these precision nutrition approaches into solutions? What are the pitfalls to avoid?
4. What are the top 2 immediate next steps towards proof-of-concept as a result of this discussion?
**Topic II: Precision Nutrition - nutritional products, supplements, and treatments**
1. What nutritional interventions or efforts are currently being practiced to mitigate nutritional challenges? What is missing from current efforts to solve this challenge?
2. Which precision nutrition approach or product could help mitigate these challenges?
3. What key steps are necessary to turn these precision nutrition approaches or treatments into solutions? What are the pitfalls to avoid?
4. What are the top 2 immediate next steps towards proof-of-concept as a result of this discussion?
